# Non-Invasive Wearables in Pediatric Healthcare: A Comprehensive Review of Uses and Implications

**DOI:** 10.3390/children12091233

**Published:** 2025-09-15

**Authors:** Kyra-Angela Magsayo, Seyedeh Fatemeh Khatami Firoozabadi

**Affiliations:** School of Engineering and Computer Science, Department of Bioengineering, University of the Pacific, Stockton, CA 95211, USA; k_magsayo@u.pacific.edu

**Keywords:** wearable technology, children, developmental delay, autism spectrum disorder

## Abstract

Wearable technology is rapidly evolving, with increasing efforts to integrate a wide range of sensors capable of capturing real-time physiological and behavioral health data from users. These devices have shown significant promise in supporting health monitoring and promoting well-being by providing continuous, objective feedback based on data analytics. Importantly, they enable early detection of potential health issues, allowing for timely intervention and more personalized healthcare. While a wide variety of commercially available wearable devices are designed for adults—tracking metrics such as physical activity, heart rate, body temperature, electrocardiograms (ECG), and oxygen saturation—there remains a notable gap in the availability and development of wearable technologies specifically tailored to the pediatric population. This narrative review paper focuses on non-invasive wearable technologies developed for individuals under the age of 18, with an emphasis on health-related applications. We examine the current landscape of pediatric wearable research, including devices aimed at monitoring developmental progress and chronic health conditions. Particular attention is given to the limited research on wearables for younger children, where physiological and developmental differences pose additional challenges. Furthermore, we explore emerging applications, identify key barriers to adoption, and discuss opportunities for future development, including improvements in design, data privacy, and age-appropriate functionality.

## 1. Introduction

Wearable biosensor devices have emerged in healthcare and are advantageous in that they allow for continuous monitoring of physiological signals and automated detection of behaviors using machine learning algorithms. These devices are non-invasive and health-related, often worn on the wrist or integrated into clothing. Multiple studies have been performed to evaluate whether the data collected from developed wearable devices is similar to the data collected in a hospital-like setting. Various data can be collected, such as heart rate, blood oxygen levels, movement, and sleep patterns. The real-time data provided by these devices increases convenience for patients and can detect and diagnose diseases early. Although wearable biosensors have been extensively studied in adult populations, their applications within pediatric populations represent a critical area of ongoing research. However, wearable biosensors for pediatric populations present distinct challenges compared to those for adults, including the need for significantly smaller devices, ensuring comfort and compliance, accommodating rapid growth, and addressing varying physiological characteristics across different age groups. This paper aims to provide a comprehensive review of wearable devices designed for children and discuss the existing challenges associated with their use.

The collection of data in real-world environments can transform the field of child development. Therefore, it is important to assess how devices are experienced by parents and children. Common concerns associated with these devices include the difficulty of donning and removing them, potential discomfort during sleep, and privacy concerns related to the inclusion of microphone sensors [1]. However, the general consensus regarding wearable biosensor devices is that people find wearable biosensors appealing due to their convenience, while at the same time maintaining the accuracy of physiological signal readings comparable to those obtained in clinical environments. The accuracy of wearable biosensor data has been explored in various studies. A notable example of this is that vital signs continuously measured with the Everion Biofourmis multi-sensor wearable device were deemed valid and reliable when the collected data was very similar to conventional measurements [2].

There are specific healthcare applications of wearable biosensor devices, including cardiovascular health, psychiatric conditions, pediatric development, and neurodevelopmental monitoring. Various designs of wearable biosensor devices for pediatric cardiovascular applications have been developed to detect and diagnose related diseases early using ECG and oxygen saturation data points [3]. Examples of psychiatric applications include remotely diagnosing and managing psychiatric disorders such as obsessive–compulsive disorder [4], integrating artificial intelligence into these devices to increase accuracy by comparing data to known patterns [5], and predicting aggressive behavior in children with autism [6]. Wearable biosensor devices are also helpful in the monitoring of child development. An important example of this is its application to ensuring children’s safety by alerting parents or guardians when the child experiences times of distress [7]. These devices have been used to provide feedback for figure skating athletes [8], as well as measuring social engagement of young children in education settings [9]. Neurodevelopment applications include the development of a wireless, skin-interfaced biosensor for cerebral hemodynamic monitoring in pediatric care [10], and the earlier detection of the onset of seizure events [11]. Based on a review of relevant patent literature, most wearable device patents—particularly those concerning wrist-worn devices such as Wearable Wrist Joint-Action Detectors and Wearable Wrist Inhalers—claim that their products can be sized and adjusted to fit both adults and children [12,13,14].

However, research and specific products in the market indicate that, in many cases, a specialized design is required to effectively accommodate the unique needs of younger children. Recent research in the field highlights the importance of these devices and identifies their future developments and applications. In this review paper, we explore the development and healthcare applications of non-invasive wearable devices specifically designed for pediatric populations. This focus is motivated by the unique challenges associated with young children, who often cannot effectively communicate symptoms or health concerns, making early detection and continuous monitoring essential. Emphasis is placed on the existing challenges, including specific hardware sensors, data privacy, ethical considerations, and regulatory constraints, which are critical factors in the design, validation, and clinical integration of such technologies. In the first part of the paper, non-invasive wearable biosensors for adults and children are explained, and the different requirements of wearable devices for these two populations are discussed. In Section 3, the importance of wearable devices is discussed, followed by a review of wearable devices for children, organized by their respective applications. Recognizing the critical importance of safety and regulatory considerations for minors, these topics are discussed in detail in Section 4. Section 5 provides a brief overview of artificial intelligence and machine learning techniques relevant to pediatric applications. As these techniques are widely applied across various domains beyond pediatrics, a detailed discussion is beyond the scope of this narrative review. Finally, we discuss applications that have not yet been specifically explored for children.

## 2. Non-Invasive Wearable Biosensors for Adults Versus for Children

Many non-invasive wearable biosensors exist for the adult population, with popular devices on the current market including smartwatches and fitness trackers, wearable rings, and smart glasses. These biosensors span fitness, health, stress monitoring, and data collection. The most common consumer-grade biosensors currently are smartwatches and fitness trackers. Popular models are the Apple Watch and the Fitbit [15,16]. Fitbit devices detect heart rate, blood oxygen saturation, skin temperature, current location, and activity. Apple Watch devices do all of these with the addition of ECG for heart monitoring. The main wearable ring is the Oura Ring [17]. This ring allows for discrete and continuous health monitoring and includes sensors for heart rate, skin temperature, and sleep. An emerging category of non-invasive wearable biosensors is smart glasses. The two main smart glasses devices on the market are the Solos Smart Glasses and Meta × Ray-Ban Smart Glasses [18,19]. The Solos Smart Glasses do not have internal biosensors of their own, but are designed to pair with real biosensors, such as smartwatches. They can display biosensor data such as heart rate, but only if the data comes from external devices, since the device itself does not track health metrics. This device connects to the downloadable Solos AirGo^TM^ app AI companion on the user’s cellular device. The Meta × Ray-Ban is good for voice AI, photography, and audio, but it also does not have its own biosensors. Therefore, it is more of a smart lifestyle assistant.

However, although many non-invasive biosensors for adults (individuals ages 18 and above) exist, not many are available for children due to the anatomical, physiological, mental, behavioral, and lifestyle differences between these two populations. There are anatomical differences between children and adults, with children still developing physically while adults have reached physical maturity. Children have ongoing growth in height, weight, and brain structure. Their brains, especially the prefrontal cortex responsible for decision-making, are still structurally immature. In contrast, adults have fully developed musculoskeletal and organ systems. Physiologically, children have, on average, a faster heart and respiratory rate than adults. Especially in early childhood, children have immature immune systems and unstable hormonal systems before puberty. Adults, therefore, have more regulated metabolism and stress responses. Children face the challenges mentally of their cognitive development still ongoing, as well as having less emotional regulation. On the other hand, adults are typically capable of abstract thought, complex reasoning, more effective emotional regulation, and better developed memory and attention span. There are also behavioral differences between adults and children. The behavior of children is strongly influenced by their immediate environment and basic needs, such as fatigue and hunger. They are more impulsive and egocentric. Adult behavior is shaped by internal motivation, personal goals, and social norms. Adults are more capable of delayed gratification and self-control. Lastly, the lifestyle differences between adults and children are also evident. The lifestyle of children is often highly structured, typically involving school and adult supervision. They have limited autonomy and depend on caregivers for basic needs and decision-making. Adults usually live more independent lifestyles and are responsible for their own personal care. They have more scheduled routines and have greater autonomy, which also comes with higher levels of responsibility and stress.

These differences between adult and child populations underscore the importance of developing non-invasive wearable biosensor devices that are catered towards specific populations, since the effectiveness and application of wearable biosensors can differ significantly across age groups. An example of this is Laarhoven’s study on how non-invasive wearable technology can be used to assess anxiety-related physiological states in young adults with autism spectrum disorder (ASD) and intellectual disabilities (ID) [20]. The Spire Stone respiration device collected real-time physiological data that correlates with changes in stress levels. It helps caregivers and educators identify specific environmental triggers for anxiety in adults who have limited verbal skills or difficulty articulating their emotional states. However, this approach does not translate well to behavioral assessment and anxiety management in children. The Spire Stone is calibrated using adult physiological baselines such as respiration patterns. Young children have very different physiological norms. Additionally, the signs of autism in children manifest as delays or deviations in social, emotional, and communicative development, rather than anxiety and stress, like in adults. The Spire Stone does not take into consideration the developmental early markers of autism, such as a lack of pretend play. This highlights the significant need for future research on developing non-invasive wearable biosensors for children.

## 3. Importance of Wearable Technology for Kids

Wearable technology for kids has various applications, including monitoring child development and improving pediatric healthcare. Using GPS sensors, wearable devices can be used to ensure safety for children by providing emergency alerts and location data for their caregiver [7]. Moreover, a heartbeat sensor in the device detects the child’s heart rate and regularly sends the data to the child’s guardians. If accelerometer sensors detect that the child suddenly falls, the child’s guardians will also be alerted. Multiple sensors are utilized in the wearable biosensor to send the child’s guardians’ safety data for the purpose of helping the guardians have a sense of security. Data from wearable technology can also be used in children’s sports to prevent injury and tailor sports drills towards improving specific skills. The use of wearable sensors is especially helpful in ensuring sports safety in children with Autism Spectrum Disorder (ASD) [21]. Children with ASD face different challenges when engaging in sports activities due to difficulties with motor coordination and sensory sensitivities. A variety of research studies performed regarding this topic have shown that wearable sensors can monitor physiological signals such as heart rate variability and biomechanical signals such as movement patterns to detect early signs of distress or potential injury. The sensors enable real-time alerts for caregivers. These features are key for improving safety in sports for children with ASD and reducing participation barriers. This application of wearable sensors is still developing, with main concerns being usability of the device, data privacy, and further research needed to validate the technology’s effectiveness [21]. Several commercial wearable devices currently exist for kids, such as the Apple Watch [16], Fitbit [15], and PocketFinder [22].

The Apple Watch is a popular wearable wrist device that has many applications for children ages 6 and up [16]. The family setup feature allows parents to set up their child’s Apple Watch without needing an iPhone and controls who the child can communicate with. Other features include activity and fitness tracking, which include digital rewards for reaching exercise milestones, real-time location tracking, emergency SOS safety features to call emergency services and contacts, health monitoring, and educational learning apps to learn new skills.

Another similar wearable wrist device currently on the market is the Fitbit ACE LTE, which is recommended for children older than 6. This device allows the user to call, text, and send voice messages for up to 20 people, allowing parents to contact their kids anytime using the Fitbit ACE app [15]. The app also utilizes advanced location technology powered by Google to allow parents to know the real-time location of the user. Other main features include an activity monitoring feature called the Noodle that records steps, jumps, and bounces, Fitbit Arcade games to reward kids for physical activity, and programmable downtime sessions for when the child is in school classes.

Smart devices are commonly worn on the wrist, but there are also smart devices that are carried rather than worn, placed in a pocket, bag, or other personal items. The PocketFinder, which is recommended for children four years old and older, is a product that can be placed in the child’s pocket or backpack and uses GPS (Global Positioning System) and GSM (Global System for Mobile Communications) to send a signal to the PocketFinder servers every two minutes [22]. The device also includes a ‘tap alert’ function, which allows the user to send an emergency message by tapping the device on a hard surface three times. Parents are able to log into the PocketFinder app to access their child’s location.

In addition to the wearable devices currently available on the market for children, research groups are actively working on wearable devices for children’s healthcare applications. Wearable biosensors for children in healthcare have been researched and developed to monitor various physiological parameters in real-time, which is especially important in the detection and diagnosis of various diseases. Some of the studies have been performed to design devices for pediatric cardiovascular applications, predict obsessive–compulsive disorder events from physiological signals, remotely diagnose and manage psychiatric disorders in children and adolescents, enhance the quality of care of pediatric patients, and predict imminent aggressive behavior before it occurs in inpatient youths with autism. The continuous data has been deemed valid and reliable through its comparison to conventional measurements [2].

**Figure 1:**
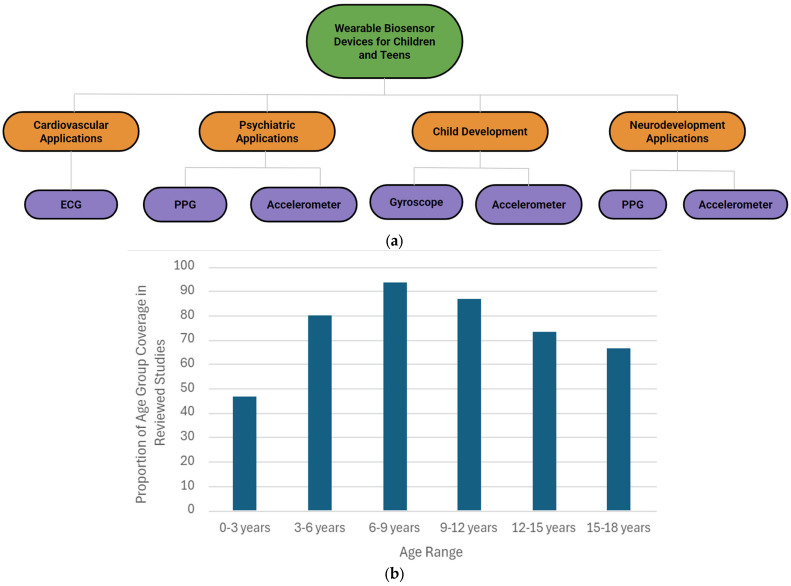
(**a**) Categorized wearable devices for children and teens based on their application. (**b**) Age Group Coverage in Reviewed Studies listed in Table 1.

Wearable devices can be categorized according to their specific applications. This paper provides a comprehensive review of various types of wearable sensors, organized by application, and concludes with an overview of the wearable sensors specifically developed for children. We focus on wearable devices for children and adolescents, with applications in cardiovascular disease, psychiatry, child development, neurodevelopment, and assistive technology, as shown in Figure 1 and Table 1. Furthermore, Figure 1a illustrates the sensors utilized for specific applications. In cardiovascular applications, ECG electrodes are commonly utilized to monitor heart activity and detect abnormalities such as arrhythmias. In psychiatric and neurodevelopmental applications, PPG (photoplethysmogram) and accelerometer sensors are widely employed to assess physiological responses, such as heart rate variability and movement patterns, which are crucial for understanding mental health conditions and tracking developmental progress. These sensors play a key role in providing continuous, real-time data that enhances the accuracy and effectiveness of clinical assessments. In the field of child development, research has predominantly concentrated on the use of gyroscopes and accelerometers. These sensors are critical for monitoring various aspects of motor development, movement patterns, and physical activity in children. Their ability to provide real-time, objective data has made them invaluable tools for assessing developmental milestones and identifying potential delays or abnormalities in motor skills. Additionally, a comprehensive list of the reviewed studies, categorized by application and targeted age group, is presented in Table 1. Moreover, Figure 1b presents the proportion of reviewed studies that concentrate on wearable devices for children, categorized by different age groups. Based on age ranges, fewer studies have focused on young children (ages 0–3 years). This underrepresentation may be attributed to several factors, including the practical and ethical challenges of conducting research with infants and toddlers, difficulties in obtaining reliable physiological or behavioral data from this age group, and limitations in the design and usability of wearable devices for very young children. Enhancing ethical standards, improving reliability, and optimizing design to align with children’s developmental stages and physiological needs are critical steps toward overcoming current challenges and maximizing the benefits for children, their families, and the broader society. Table 1 includes a detailed summary of all 15 studies that utilize wearable devices for various applications aimed at children and adolescents. It outlines the objective of each study, the type of device used, the sensors employed, and the age range of the users involved.

### 3.1. Cardiovascular Applications

The ability of wearable biosensors to gather continuous physiological data in real time has the potential to make major advances for patients with chronic diseases, such as congenital heart disease [3,22,23,24]. These devices can be implemented to detect early signs of clinical deterioration through the detection of specific patterns known to precede certain conditions. Mobile technology is commonly used in adult cardiovascular populations, but not so much in pediatric populations. Specific pathologies in pediatric cardiology can benefit from wearable biosensors, such as single-ventricle heart disease. Patients who are between the first and second stages of surgical palliation have high morbidity and mortality. Continuous physiological data could be used to predict cardiopulmonary arrest occurrences when the patient is at home. The second and third stages of this disease can be monitored using the oxygen saturation data points from the wearable biosensors. Another possible use of wearable technology is the prevention of adulthood atherosclerotic cardiovascular disease. Step counting technology can also be integrated to encourage patients to increase physical activity and weight loss. However, developing wearable biosensors for the pediatric population has its specific challenges.

**Table 1:** A list of reviewed research studies involving minors.

Study	Device	Wearable Sensor	Age (Years)
Evaluating Users’ Experiences of Child Multimodal Wearable Device: Mixed Methods Approach [1] (2024)	Little Beats	audio, electrocardiogram, motion sensors	0–9.5
Wearable Safety Device for Children [7] (2022)	Custom device	GSM, GPS, temperature sensor, accelerometer, heartbeat sensor, and panic button	0–18
Wearable Biosensors in Pediatric Cardiovascular Disease [3] (2019)	wearable Biosensors	ECG and oxygen saturation data points, pulse oximetry sensors	0–18
Training, children, and parents: Coach perspectives on wearable sensor data in sub-elite figure skating in the United States [8] (2023)	Wearable inertial measurement unit (IMU) sensors	gyroscope, accelerometer	4–18
Initial validation of wearable sensors to measure social engagement of young children [9] (2022)	Custom wearable engagement sensor badges worn in the pocket of a custom-made t-shirt	Accelerometer, gyroscopes, ultrasound	3–4
Predicting Obsessive–Compulsive Disorder Events in Children and Adolescents in the Wild Using a Wearable Biosensor (Wrist Angel) [4] (2023)	E4 Empatica Biosensor Wristband	PPG, accelerometer, optical thermometer	8–16
Use of Mobile and Wearable Artificial Intelligence in Child and Adolescent Psychiatry: Scoping Review [5] (2021)	ECG strap and wrist-worn biosensor	accelerometer, gyroscope, magnetometer	0–18
Wearable Technologies for Pediatric Patients with Surgical Infections-More than counting steps? [2] (2022)	Everion by Biofourmis multi-sensor wearable device	PPG, infrared sensor, temperature sensor	4–17
A wireless, skin-interfaced biosensor for cerebral hemodynamic monitoring in pediatric care [10] (2020)	Custom wireless device	multiwavelength reflectance-mode PPG, functional near-infrared sensors	0.2–15
Longitudinally tracking personal physiomes for precision management of childhood epilepsy [11] (2023)	Microsoft Band	PPG, accelerometer, GSR, gyrometer, GPS, microphone, UV sensor, skin temperature sensor, barometer	0–18
Wearable Biosensing to Predict Imminent Aggressive Behavior in Psychiatric Inpatient Youths with Autism [6] (2023)	E4 Empatica Biosensor Wristband	PPG, accelerometer, optical thermometer	5–19
Long-term gait postural characteristics of children with general foot pain using smartphone-connected wearable sensors [25] (2025)	Wearable inertial measurement unit (IMU) sensors	Accelerometer, gyroscope	7–11
Wearable electromyography recordings during daily life activities in children with cerebral palsy [26] (2020)	wearable leggings with embedded textile EMG electrodes	Electromyography (EMG)	6–13
Smartwatch Measures of Outdoor Exposure and Myopia in Children [27] (2024)	Smartwatch	Light sensor	6.6–7.8
Towards Continuous Social Phenotyping: Analyzing Gaze Patterns in an Emotion Recognition Task for Children with Autism through Wearable Smart Glasses [28] (2020)	A prototype of Google Glass glasses learning aid called *Superpower Glass*	Infrared eye tracker, accelerometer	6–17

Unlike adults, the physiological parameters of children are very broad in range. The heart rates of healthy infants can be as high as 180 beats per minute, and patients with single-ventricle diseases have oxygen saturation values of approximately 70–80% [3]. Thus, wearable biosensors for children must be optimized to handle extreme physiological ranges. This must take into consideration the accuracy, size, cost, battery life, data security, and comfort of the device. Socioeconomic factors can significantly affect children’s access to wearable technology, influencing not only whether children can obtain these devices but also how effectively they can use them. In general, children need to use the devices under the supervision of parents and usually with the interface application on smartphones, which may not be applicable for families with a lack of digital literacy and limited access to financial resources. Another challenge is correctly choosing pediatric populations for clinical testing. Healthy children usually have low occurrences of catastrophic health events and low mortality rates. These populations will have low positive predictive values due to the low occurrences. This will result in more false positives than false negatives. Because of this, high-risk populations should be used instead of healthy populations.

The accuracy and reliability of wearable biosensor data in pediatric populations have been evaluated in clinical settings to ensure their effectiveness in patient monitoring. A pilot study investigated the quality and accuracy of physiological signal data collected through the continuous telemonitoring of a pediatric surgical ward of the University Children’s Hospital Basel, Switzerland. The data of 21 pediatric patients with an average age of 9.1 years were collected and monitored from December 2018 to July 2019 [2]. The subjects were hospitalized for the conditions of appendicitis, septic arthritis, or osteomyelitis. Their vital signs were measured with the Everion Biofourmis multi-sensor wearable device. Analysis of the data revealed that the heart rate and oxygen saturation data measured by the wearable device were in strong agreement with the same data type collected by nurses in the hospital. However, the temperature readings were lower than the actual, but this was attributed to the use of different measurement techniques [2].

In addition to ensuring the accuracy of wearable biosensors, their design must also prioritize the comfort of pediatric patients. The design of wearable devices should allow the cardiovascular pediatric patients to feel and act like kids, as opposed to constantly monitored patients. The wearable biosensors worn by adults would need to be modified for children, in that they need to be smaller in size and account for the rapid physical growth that occurs in childhood development. Adhesive sensors, such as patches, are an alternative but can be limited by skin sensitivity to the adhesive.

### 3.2. Psychiatric Applications

Wearable devices have found several important applications in the field of psychiatry, particularly in the monitoring and management of mental health conditions. For instance, these devices have been used to predict and detect events associated with obsessive–compulsive disorder (OCD) by tracking physiological and behavioral markers [4]. In addition, wearable technology has shown promise in identifying and anticipating imminent aggressive behaviors in inpatient youth with autism spectrum disorder, enabling timely interventions and improved management of these patients [6]. Furthermore, the integration of artificial intelligence (AI) with wearable devices has opened up new possibilities for the remote diagnosis, monitoring, and management of various psychiatric disorders. AI-powered wearables are capable of analyzing real-time data, providing clinicians with actionable insights, and enabling personalized care strategies for individuals with mental health challenges [5]. This technological convergence holds great potential for improving outcomes and enhancing the overall efficiency of psychiatric care.

**Figure 2:**
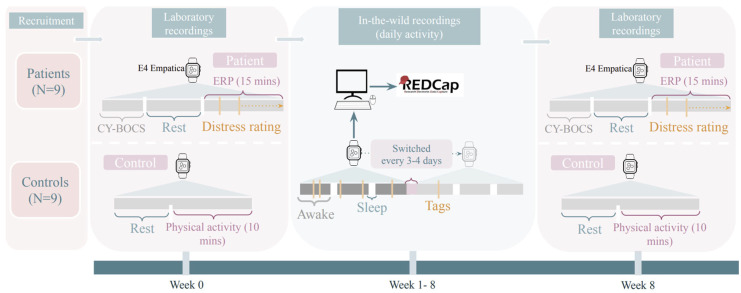
E4 Empatica device and method for recording data in an experiment predicting obsessive–compulsive disorder events. The following are the meanings of the abbreviations used: CY-BOCS (Children’s Yale Brown Obsessive–Compulsive Scale), ERP (Exposure and response prevention), OCD (Obsessive-Compulsive Disorder), REDCap (Research Electronic Data Capture) [4]. Figure 2. Reprinted from [4] ©Kristoffer Vinther Olesen, Nicole Nadine Lønfeldt, Sneha Das, Anne Katrine Pagsberg, Line Katrine Harder Clemmensen. Originally published in *JMIR Res Protoc. (2023)* https://www.researchprotocols.org/2023/1/e48571 (accessed on 30 July 2025) under a Creative Commons CC BY-NC-ND 4.0 License (https://creativecommons.org/licenses/by-nc-nd/4.0/). No changes were made.

Physiological signals recorded using the wrist-worn E4 Empatica device were studied to evaluate the feasibility of predicting OCD events [4]. The eighteen participants were youths aged eight to sixteen years and wore the wrist biosensor for eight weeks. Nine of the participants had an OCD diagnosis, while the other nine did not. The participants were instructed to press a button on the device when they were stressed by OCD symptoms or whenever they felt very scared. The participants met with the researchers every three days to switch the wristbands and gather biosignal data. The data was transferred to the hospital server. This procedure is illustrated in Figure 2 (Reprinted from [4], ©Kristoffer Vinther Olesen, Nicole Nadine Lønfeldt, Sneha Das, Anne Katrine Pagsberg, Line Katrine Harder Clemmensen. Originally published in *JMIR Res Protoc. (2023)* under a Creative Commons CC BY-NC-ND 4.0 License (https://creativecommons.org/licenses/by-nc-nd/4.0/).

The E4 Empatica Biosensor Wristband used in this study contains multiple components, including a photoplethysmographic (PPG) sensor, an EDA sensor, a 3-axis accelerometer, an optical thermometer, and an event tag button that records a point in time when pressed. The PPG sensor uses infrared light to measure changes in blood volume (Blood volume pulse, BVP), while the EDA sensor measures skin conductance sampled 4 times per second. The results of the study were that most patients had moderate to severe OCD, and 1 had mild. However, the limitations of the E4 Empatica Biosensor Wristband include the physiological signals containing high levels of noise and artifacts, and some data loss. These factors negatively affect the overall reliability of the wearable device.

Another study highlights the use of the E4 Empatica Biosensor Wristband in predicting imminent aggressive behavior in psychiatric inpatient youths with autism [6]. In this study, performed by Imbiriba et al., it was investigated whether changes in peripheral physiology recorded by a wearable biosensor and machine learning can be used to predict imminent aggressive behavior before it occurs in inpatient youths with autism. This study involved 70 study participants aged 5–19 years old. Inpatient study participants with autism wore the commercially available and regulatory-compliant E4 biosensor on their non-dominant wrist. The research team conducted observations with minimal disruption to the participants’ daily inpatient routines, which encompassed academic lessons, various therapies—including behavioral, occupational, speech, and milieu therapies (psychotherapy within therapeutic communities)—as well as meals and recreational periods. A total of 429 naturalistic observational coding sessions were recorded, totaling 497 h, wherein 6665 aggressive behaviors were documented, including self-injury, emotion dysregulation, and aggression toward others.

Mobile and wearable artificial intelligence (AI) has also been integrated into the evaluation of psychiatric disorders [5]. There is growing evidence of large data-driven approaches, such as AI, being beneficial in that physicians are able to individualize diagnoses and treatment management of psychiatric disorders in adults. The possibility of applying this to child and adolescent psychiatry is being explored. Researchers have identified areas of innovation with wearable devices that are conducive to artificial intelligence applications to remotely diagnose and manage psychiatric disorders in children and adolescents. In a specific study performed by Welch et al., child and adolescent psychiatric patients aged 0–18 years were test subjects instructed to wear an ECG strap and wrist-worn biosensor. The biosensor monitored heart rate and sleep and included an accelerometer. The data was used to detect behavioral changes in patients and was found to accurately predict the onset of behavioral changes related to psychiatric disorders.

### 3.3. Child Development

Wearable biosensors have also been used to measure social engagement among young children in education settings [9], which is an important indicator of the level of child development. Social engagement involves the interaction between two or more individuals engaging in verbal or nonverbal behaviors. Children with disabilities such as autism, speech and language impairments, and learning disabilities are at risk for social challenges. Moreover, children without disabilities may have social skill deficits if they do not often engage in social interaction. Not having social skills places children at risk for anxiety or depression, which may then lead to difficulties in social adjustment during adolescence and adulthood. It is important for early childhood educators to accurately measure the social skills of their students in order to provide direct support to children to develop social skills. This can also improve the effectiveness of class instruction.

In the other study performed by Douglas et al., wearable engagement sensor badges were worn in custom-made t-shirts by children ages 3 to 4 [9]. The sensors were designed to detect frontal orientation, physical activity levels, proximity of communication partners, and speech in the environment. A wearable networked sensor system called WES (Wearable Engagement Sensor) was developed and used in this study. The WES badges were custom-made using commercially available components such as micro-controllers and accelerometers. These badges were worn by the child and teacher participants. An integrated sensor-based station collected data from the badges, and the user interface provided visualization of the data in real-time in the form of line graphs. A 400 MHz radio link was used to transfer the data from the badges to the wireless base station. The design of the WES allowed the children to participate in their normal classroom activities while simultaneously collecting social engagement data. WES uses ultrasound sensing to capture interactive modalities, which mainly include the occurrence and duration of frontal orientation (when two individuals face each other) and proximity (physical distance between two individuals measured in centimeters). The sensor data was compared to video-coded data for social engagement between child-child and teacher–child. The aim of the experiment was to have 85% reliability between the data [9].

Another application of the wearable devices for child development is the use of wearable inertial measurement units (IMU) sensors in children aged 4 to 18 years to gather motion data in sub-elite figure skating [8]. Sub-elite figure skating refers to athletes who have mastered advanced skills but are not yet competing at the elite international level. This level of competition was chosen because there are more athletes at the sub-elite level than the elite level, allowing for a greater impact on more people if the data were found to be beneficial. IMU data can help prevent injury and improve performance by minimizing the risk of overuse injuries while maximizing the intensity of training.

The amateur athletes worked closely with the recruited eight coaches, who must have coached at least 1 skater who competed at the sectional level in the past 5 years. The coaches in the study had at least 15 years of coaching experience and were in the United States. Coaches were chosen to be interviewed because they have more experience than child-athletes since they often work with many over the years. This made interviewing coaches a better starting point for understanding how IMU data could be used in figure skating training. An assessment of how coaches felt about sharing the IMU data with the athlete and parent was performed to determine if IMU data should be more broadly adopted in training. Consideration of sharing information with the athlete’s parents and family was performed because they are invested in training outcomes since they provide financial and logistical resources (for example, transportation to practice) for the athletes [8]. The main opinions collected from the coaches were that sharing data could actually be detrimental to the athlete’s performance and should therefore be minimized because sharing data can cause unhealthy competitiveness among athletes, and parents may push to have the coach only practice a certain skill or may downplay the severity of the athlete’s injury. This study highlighted the importance of privacy concerns regarding the data collected by various wearable biosensor devices. It raises the concern over who should have access to the data and when. Data sharing must be limited if sharing leads to negative outcomes, such as data misuse.

A further application of wearable biosensors examines how gait characteristics evolve as children grow and how the foot may indicate underlying musculoskeletal issues [24]. Through childhood, children’s gait patterns are still maturing, so analyzing their walking posture using accelerometer and gyroscope sensors (IMUs) provides insight into normal and abnormal foot mechanics. A study found that children with foot pain exhibited increased rearfoot angle and reduced toe-off coronal movement, which could affect propulsion and weight transfer during walking. The IMU data revealed that gait abnormalities were more prominent in the left foot, suggesting potential laterality effects in foot pain development [24]. This study suggests the use of wearable biosensors for early detection of gait abnormalities, which could assist in diagnosing and preventing musculoskeletal disorders in children.

There is another study that aimed to evaluate the potential of the Language Environment Analysis (LENA) system as a clinical research tool for autistic toddlers. The study involved 100 autistic children aged 16 to 33 months and their caregivers. A wearable recording device, placed in a front-facing pocket of a vest worn over the child’s clothing, was used to capture up to 16 h of naturalistic audio from the children and their environments. The study focused on three LENA-derived metrics: Conversational Turn Count (CTC), Vocal Productivity (VP), and Automated Vocalization Assessment (AVA). These measures demonstrated small-to-moderate, yet significant positive correlations with established clinical language assessment variables, suggesting their potential utility in early language monitoring. Despite these promising findings, several limitations were noted. First, the sample was limited exclusively to autistic children, restricting generalizability. Additionally, many participants exhibited minimal spoken language, likely contributing to floor effects in clinical assessments, which may underestimate the true validity of LENA variables. Furthermore, the LENA system analyzes only segmented language features and does not provide semantic or grammatical analysis, which may lead to the misclassification of certain “conversational turns”. The cross-sectional study design also limits conclusions about the predictive value of these metrics for future language development. Importantly, the data collected were not used to detect or diagnose any health conditions, but rather to explore associations between LENA variables and standardized language measures in autistic children [29].

### 3.4. Neurodevelopment Applications

**Figure 3:**
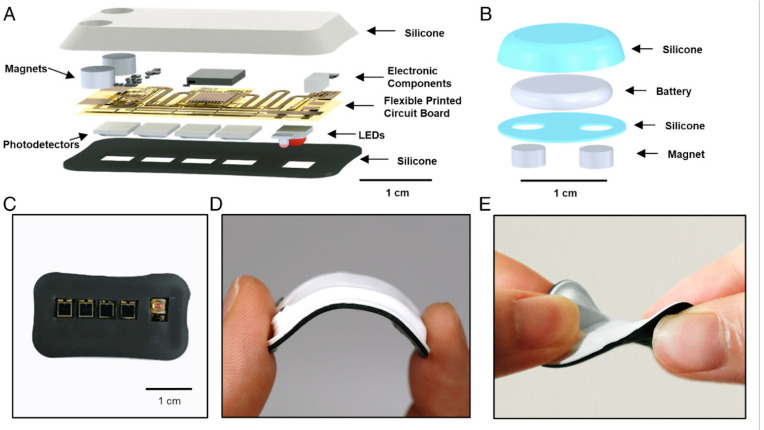
Design and Mechanical Testing of a Soft, Wireless Device for Pediatric Cerebral Hemodynamic Monitoring. (**A**,**B**) Schematic and exploded views of the device with a modular, rechargeable coin cell battery. The flexible design includes serpentine interconnects linking electronic components for wireless operation and optical sensing, with four photodiodes at varying source–detector distances. Magnetic connectors enable secure attachment, all encapsulated in medical-grade silicone. (**C**) Skin-facing side showing LEDs and photodetectors between a black optical barrier and transparent PDMS for optimal light coupling. (**D**,**E**) Photos of the device under (**D**) bending and (**E**) twisting stress [10]. Figure 3 Reprinted from ©Alina Y. Rwei et. al. (2020), *Proceedings of the National Academy of Sciences (PNAS),* https://www.pnas.org/doi/10.1073/pnas.2019786117 [10], under a Creative Commons CC BY-NC-ND 4.0 License (https://creativecommons.org/licenses/by-nc-nd/4.0/). No changes were made.

Wearable biosensor devices have emerged as a transformative tool in healthcare, particularly in neurodevelopmental applications. These devices leverage advanced sensor technology to monitor various physiological parameters in real-time, offering valuable insights into a patient’s health as well as monitor muscle tone activity in daily life for children with Cerebral palsy [11,25]. A recent and significant advancement in neurodevelopmental applications of wearable biosensor devices is the creation of a wireless, skin-interfaced biosensor specifically designed for cerebral hemodynamic monitoring in pediatric care [11]. This innovative biosensor enables real-time, non-invasive monitoring of blood flow and oxygenation levels in the brain, offering valuable insights into cerebral health and function. Particularly beneficial for pediatric patients, the device provides continuous monitoring without the need for invasive procedures, reducing the risk of complications and discomfort. By offering a more accessible and efficient method for tracking cerebral hemodynamics, this wearable technology has the potential to greatly improve early detection and management of neurological conditions in children, enhancing both patient outcomes and overall care strategies [11]. A clinical study on pediatric subjects aged 0.2 to 15 years and with various racial backgrounds was performed with the objective of enhancing the quality of care of pediatric patients, particularly those at risk for cerebral and neurodevelopmental impairments. A custom-made wireless, miniaturized, and mechanically soft, flexible device was developed. The details of the hardware components are shown below in. Figure 3 reprinted from ©Alina Y. Rwei et. al. (2020), *Proceedings of the National Academy of Sciences (PNAS),* https://www.pnas.org/doi/10.1073/pnas.2019786117 [10], under a Creative Commons CC BY-NC-ND 4.0 License (https://creativecommons.org/licenses/by-nc-nd/4.0/). The miniature size of this device allowed placement on any location on the heads of pediatric subjects, with the only limitation being regions with dense hair growth. Multi Photodiode array and light-emitting diodes were incorporated into the device to simultaneously monitor systemic and cerebral hemodynamics. The cerebral oxygenation, heart rate, peripheral oxygenation, and cerebral vascular tone were monitored [11].

The overall layout of the developed device included a flexible printed circuit board with two main units: (1) a Bluetooth low-energy (BLE) system on a chip (SoC) module (nRF52832; Nordic Semiconductor) with associated components for power regulation and wireless communication, and (2) an optical sensor for continuous monitoring of systemic and cerebral hemodynamics. The device was encapsulated with a medical-grade silicone elastomer, resulting in a compact device with dimensions of 33 × 16 × 3 mm and a mass of 2.8 g. This was estimated to be an order of magnitude lighter than NIRS (Near-infrared spectroscopy) probes currently used in the clinic. The data is passed wirelessly from the sensor to the BLE-enabled device, such as a tablet computer or a smartphone, for real-time data storage and display.

The results of the study were that the device provided high-precision measurements comparable to clinical measurements. Cerebral oxygenation, heart rate, and arterial blood oxygenation data were validated against medical-grade devices, showing minimal deviation from the standard clinical measurements. Moreover, the biosensor’s ability to track physiological responses was validated through its detection of significant decreases in cerebral oxygenation in children with congenital hypoventilation syndrome (CCHS) and through its recording of increased cerebral oxygenation in the hyperoxia tests, where subjects inhaled 100% oxygen. No adverse skin conditions were observed from the device. The wireless design minimized discomfort in the subjects and allowed them to move freely. This indicated that the device was safe for continuous use and reduced the stress associated with constant hospital monitoring [11].

Seizure onset detection is a crucial component in managing neurological disorders such as epilepsy, particularly in children, where early intervention can greatly reduce risks and improve long-term outcomes. In recent years, advancements in technology have enabled more precise and efficient monitoring of seizure activity. Wearable biosensors have also been implemented into a real-time cloud computing infrastructure to integrate wearable sensors and machine learning to improve early detection of seizure onsets in children [11]. The wearable sensors connected to a cloud computing LOOP system allow the tracking of an individual’s personal activity and physiological signals longitudinally in order to identify clinically concerning events.

**Figure 4:**
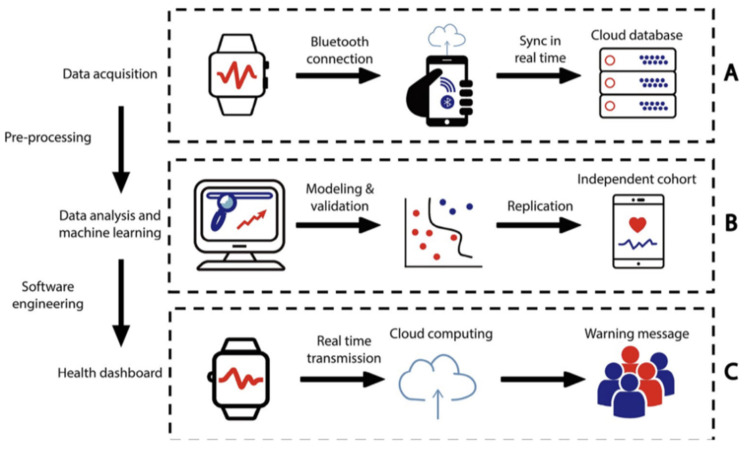
Overview of the LOOP system to detect seizure onset in epilepsy patients [11]. Using Bluetooth, the Microsoft Band wristband device was connected to a nearby smartphone. (**A**) The data was collected and stored in real time. (**B**) An installed app on the phone allowed the caretaker to record the child’s seizure events. (**C**) This data was stored in a remote cloud for analysis. This data could be accessed via a user interface on the cell phone or cloud server. Figure 4 reprinted from © Jiang et al. (2022), *PLOS Digital Health*, https://doi.org/10.1371/journal.pdig.0000161 [11], licensed under CC BY 4.0. No changes were made.

In a study performed in 2021 [11], 99 children who were diagnosed with epilepsy were recruited and tracked in time increments of one second using a wearable wristband. The wristband device was the Microsoft Band. Many data points were collected for the parameters of heart rate and stress response across age groups. This data was analyzed to identify any physiological irregularities upon epilepsy onset. The physiome and activity profiles of the children displayed patterns across the age groups, with the factors of age and sex highly affecting circadian rhythms and stress responses across the developmental stages. With the application of the machine learning framework, the LOOP system is in Figure 4. (reprinted from © Jiang et al. (2022), *PLOS Digital Health* [11], https://doi.org/10.1371/journal.pdig.0000161, licensed under CC BY 4.0.) was demonstrated to be able to detect subtle seizures and seizures prior to clinical onset by comparing data to the children’s personal physiological baseline values.

The results of this research study validated the effectiveness of using real-time mobile infrastructure in a clinical setting. Subtle seizures that were not able to be recognized by humans were able to be detected prior to clinical onset using this approach, thus proving this application very useful in the care of epileptic patients. Further considerations of clinical applications of this approach led researchers to then develop a personalized health dashboard for patients. This feature is accessible on computers or mobile devices, allowing caregivers to monitor patients’ physiological conditions in real time. The digital health platform developed by these researchers can be broadly applied to health management in general. Therefore, its use can be extended to many other diseases, such as cardiovascular and diabetic diseases.

In another study [30] researchers primarily utilized Empatica E4 Wristbands to gather electrodermal activity (EDA) data, a physiological indicator of stress. These particular wristbands were selected because they measure various neurophysiological indicators, including EDA. The study’s targeted demographic consisted of autistic children and adolescents. The age range for the participants was predominantly 5 to 18 years old. The researchers aimed to compare different methods of processing EDA data using two open-source software programs, NeuroKit2 and Ledalab, and to examine how various EDA metrics correlate with observed child behaviors. While the study found strong correlations between the same EDA metrics across the two software programs, indicating they track similar patterns, there were significant differences in the raw values they reported. The findings revealed that more frequent EDA peaks were associated with positive social behaviors, while a higher amplitude of peaks was linked to less adaptive behaviors and negative mood in autistic children during a parent–child play interaction, highlighting the complexity of using EDA as a biomarker.

### 3.5. Assistive Devices for Children

Non-invasive wearable biosensors serve a vital role in monitoring physiological data such as heart rate and movement in children. They are part of a larger, broader category of assistive wearable devices [31]. These devices encompass a wide range of functions from support for communication to cognitive function in pediatric populations. Common examples of assistive devices available on the market for children include smart glasses [28], interactive clothing, functional electrical stimulation (FES) systems, and sleep trackers. Smart glasses for children include the Envision Glasses [32] and the OrCam MyEye [33] and are mainly used by visually impaired children. Envision Glasses convert visual information into audio. The OrCam MyEye is a small, AI-powered camera attached to glasses that reads texts aloud and identifies objects and faces. There has been research performed to investigate medical applications of smart glasses. One example is a 2019 study by Haber in which gaze patterns were analyzed in an emotion recognition task to detect autism in children ages 6–17 years [28].

The Sense-ational You clothing brand [34] is an example of an interactive clothing assistive device. The clothing tops have sound and light reducing tools as well as built-in fidgets. This is helpful for children with autism or sensory processing disorder. Popular sleep tracker assistive devices include the Fitbit Ace Series and the Garmin Vivofit Jr. These trackers are designed for children to track sleep, activity, and steps. They also include family apps for parental monitoring [34]. The Octopus Watch by Joy is an educational wearable smartwatch for children that uses interactive icons to teach time management and routine-building [34]. Functional electrical stimulation (FES) mechanisms are also available for children.

Functional Electrical Stimulation (FES) is a rehabilitative technique that uses low-level electrical currents to stimulate nerves and muscles. This helps children with neuromuscular impairments improve movement [35]. A commonly used FES device is the WalkAide Pediatric System [36]. This FES system is designed for children with foot drop, which is a condition where the child has difficulty lifting the front part of the foot, leading to abnormal gait. The WalkAide device stimulates the lower muscles in children with foot drop to improve gait.

## 4. Safety and Reliability

Several studies have been performed to determine the safety and reliability of non-invasive wearable biosensors for children. In a study performed in 2018 by Pelizzo [37] on the accuracy of a wrist-worn heart rate sensing device for elective pediatric surgical procedures in children ages 4–16 years, it was demonstrated that the wearable photoplethysmography (PPG) sensors used in the Fitbit Charge HR were highly accurate in monitoring heart rate in children undergoing surgery. These physiological parameters were compared to the clinical gold standards of continuous ECG and pulse oximetry and were very similar in performance, as determined by statistical analysis. The PPG sensors were also deemed safe, with there being no posed additional risk to the patient.

Optical sensors were also found to be both safe and reliable in a study performed by Herbert in 2020 [38]. The optical sensors used (Philips IntelliVue MP70 monitors) were validated in previous studies, and a standardized protocol was followed to measure the respiratory rate in the participants. The participants ranged in age from birth to 13 years. The data collected for the respiratory rates matched known respiratory physiology in children. Furthermore, the sensors were deemed safe since they were very small, hidden under clothing, and caused very minimal distress for the participants. Optical sensor systems have also proven to be safe and reliable through performance in real schoolyard environments [39]. The participants of this study were children aged 5 to 15 years. These sensor systems utilized accelerometers and GPS and showed consistent tracking of movement, activity levels, and social interactions. The sensitivity was high enough to detect small behavioral patterns such as speed change during bicycling. Moreover, the system was not distracting or harmful to children.

In a 2024 study performed by Whitney Au, a systematic review was performed on the effectiveness of wearable activity trackers on physical activity in children aged 19 years old or younger [40]. The data was concluded to be reliable since randomized control trials were used, two independent reviewers screened the studies to reduce error and bias, and statistical analysis was performed to show that the results were consistent across different studies. The trackers had no adverse effects in regard to safety.

Studies have also been performed on the reliability and safety of wearable devices in children’s medical applications, such as depression, obesity, and neuromuscular disorders. Mobile and wearable technology has been assessed for depressive symptom monitoring in children and adolescents up to 18 years of age [41]. This application showed promising results, but still needs further validation to determine the full extent of its reliability. This review included 30 studies, each with different populations, methods, and objectives. It tested whether the mobile technology could work, rather than the reliability of the data. The technology was passive and non-invasive, posing no safety risks to the user. In a 2022 study performed by Wentao Wang [42], the effectiveness of wearable devices as physical activity interventions was tested for preventing and treating obesity in children up to 18 years of age. This review included 12 randomized controlled trials. The results were consistent across studies, with statistically significant improvements in the participants over time. No safety concerns were reported, suggesting that the wearable devices were safe for use in children and adolescents. However, the long-term effectiveness of the devices still needs to be studied. Potential and current limitations of wearable sleep monitoring technologies for children with neuromuscular disorders are highlighted in [43]. Current wearable devices rely on movement data and are effective at detecting when children are sleeping. However, the motion-based sensors are not as effective for children with limited mobility and lack validation in children with neuromuscular disorders. The author proposes improving the reliability of these devices by incorporating multimodal sensors that monitor heart rate and body temperature, as well as adding machine learning. No safety concerns were found, with these wearable devices being non-invasive and low-risk.

### 4.1. Safety Concerns of Wearable Devices

Although non-invasive wearable devices for children have increased in popularity over recent years, there remain concerns regarding screen time, privacy, and the overall health of the individual [44].

Wearable devices often include screens, which poses the concern of excessive screen time for children. Too much screen time can increase the risk of health issues such as eye strain and sleep disturbance. Since wearable devices collect personal data, including biometrics, location, and activity patterns, privacy concerns are also an issue. There is potential for this information to be misused. This is especially dangerous for children. The overall health, both mental and physical, could be negatively affected by wearable devices. Children may become overly reliant on and distracted by their wearable devices, decreasing their social interactions and connections. Fixation on the monitoring of physical activity can lead to hyper-awareness of body image. While wearable devices monitor steps taken and calories burned, these metrics alone are not indicators of holistic health and well-being. If too much of the children’s attention is on these, they may not engage in more diverse forms of physical activity and play.

However, while these safety concerns exist, wearable devices can positively support children’s health and development when used mindfully.

### 4.2. Rules and Regulations for Collecting Physiological Data Acquired from Children

There are two main regulations for protecting children’s data online. COPPA (Children’s Online Privacy Protection Act) and GDPR-K (General Data Protection Regulation for Children) are both laws aimed at safeguarding children’s privacy online. COPPA, a U.S. regulation, applies to children under the age of 13 and mandates obtaining verifiable parental consent before collecting their personal data. GDPR-K, a component of the broader GDPR in the European Union, protects children under 16 [45,46]. COPPA applies to commercial websites and online services—including mobile apps and IoT devices—that target children under 13 and collect, use, or share their personal information. It also applies to general audience platforms that know they are handling personal data from children under 13, as well as services that knowingly collect such data from users of child-directed platforms. While the COPPA regulates commercial online services, research studies involving children are subject to Institutional Review Board (IRB) approval. This process requires detailed documentation of the study protocol, including parental consent forms that outline the types of data to be collected and the methods for data storage and protection. Moreover, if the wearable device is categorized as a medical device, the Food and Drug Administration (FDA) in the USA or in Europe, under the EU Medical Device Regulation (MDR) 2017/745 has specific regulations which is committed to supporting the development of safe and effective medical devices [44,47,48]. Under these regulations, manufacturers are required to demonstrate clinical safety and performance for specific age groups, rather than relying on adult data extrapolation. This poses both scientific and ethical responsibilities for developers of pediatric wearables, particularly in relation to usability, long-term safety, and physiological compatibility with children’s developing bodies. Furthermore, the MDR emphasizes post-market surveillance and risk management, which are especially relevant for continuous monitoring devices. In parallel, device cybersecurity has become a regulatory priority, with the FDA’s premarket guidance outlining expectations for threat modeling, software lifecycle management, and real-time patching—all essential to protect pediatric users whose data are highly sensitive and prone to long-term implications if breached. Privacy regulations further reinforce the need for “privacy by design” approaches. In the UK and EU contexts, Data Protection Impact Assessments (DPIAs) are mandatory for technologies that process high-risk personal data, such as pediatric health information. The ICO’s Age Appropriate Design Code (Children’s Code) provides a practical framework for ensuring that digital health services are transparent, minimally intrusive, and aligned with the best interests of the child. These standards require developers to provide clear data flows, limit profiling, and ensure children can exercise their data rights. Meanwhile, in the U.S., regulatory distinctions between HIPAA-covered health data and consumer-generated health data (e.g., from fitness trackers or wellness apps) can create ambiguity. Pediatric wearable developers must ensure that, regardless of legal classification, robust data protections are applied uniformly. Aligning device development with both healthcare regulations and consumer protection laws is essential to earn parental trust, protect minors, and ensure regulatory compliance across jurisdictions.

## 5. Wearable Sensors for Children: AI and ML Integration

While novel hardware sensors are central to this review study, a brief overview of Artificial Intelligence (AI) and Machine Learning (ML) techniques is provided to contextualize their integration and application in healthcare data analysis. Several studies among the studies in Table 1 have demonstrated the utility of machine learning (ML) in health and behavioral sciences, particularly in pediatric and adolescent contexts. In [1], ML algorithms were employed to process audio signals, though their use raised privacy concerns among parents and guardians. Similarly, Ref. [3] described the application of ML for data compression and pattern recognition to detect cardiovascular disease in pediatric patients. In Ref. [8], a classification model based on inertial measurement unit (IMU) data was developed to assess the expertise level (“eliteness”) of athletic individuals. In other research, the anticipated benefits of continued development of the Wearable Evaluation System (WES) were discussed [9]. Researchers argued that with further refinement—through advanced ML and software algorithms—the WES could provide a more efficient, objective, and less intrusive alternative to traditional, teacher-led observation methods [9]. Meanwhile, Ref. [12] presented a machine learning framework designed to accurately detect the onset of epileptic episodes. This approach utilized statistical feature extraction in combination with a bootstrapped ensemble classifier network for effective classification. Additionally, Ref. [6] implemented ridge-regularized logistic regression, support vector machines (SVMs), and neural networks (NNs) to make real-time binary predictions of aggressive behavior using extracted time-series features. Both population models (PMs) and person-dependent models (PDMs) were updated every 15 s for dynamic decision-making. In the context of psychiatric disorders, Ref. [4] introduced generalized predictive models using one-dimensional convolutional neural networks (1D CNNs) to forecast obsessive–compulsive disorder (OCD) events. The study compared models trained solely on OCD patient data with those incorporating both patient and control subject data, analyzing differences in predictive performance. A broader overview of the field was provided in [5], where a scoping review summarized existing research on the application of AI and ML in child and adolescent psychiatry, and outlined future opportunities for advancement.

The reviewed studies highlight the growing role of machine learning in enhancing healthcare, behavioral monitoring, and educational assessment, particularly in pediatric and adolescent populations. From disease detection and behavioral prediction to context-aware sensing and automated observation systems, ML techniques demonstrate significant potential to improve accuracy, efficiency, and personalization in various domains. However, these advancements also bring challenges, such as privacy concerns and the need for rigorous validation across diverse populations. Continued interdisciplinary research and ethical considerations will be essential to fully realize the benefits of machine learning in sensitive applications involving children and adolescents.

## 6. Discussion: Importance of Developing Wearable Technology for Children for Tracking Development Milestones and Disorder Diagnosis

Lack of proper communication in children can significantly contribute to misdiagnosis, as many children, especially younger ones, struggle to express their feelings or symptoms clearly due to limited vocabulary or developmental stage. This can lead healthcare providers or educators to misinterpret behaviors or overlook critical signs of medical or psychological conditions. For example, a child may not be able to articulate physical pain, like a headache or stomach discomfort, and might display irritability or fatigue instead, which can be mistaken for moodiness or behavioral issues. Similarly, children with developmental conditions such as autism may demonstrate distress in social settings, but without understanding the underlying sensory or social challenges, these behaviors might be wrongly labeled as simple shyness or antisocial behavior.

Additionally, when children cannot effectively communicate their symptoms, it can result in missed or incorrect diagnoses, particularly with mental health or chronic conditions. Symptoms such as anxiety or depression may manifest through behavior that looks like typical childhood moods, making it hard for caregivers or professionals to recognize the underlying issue. Communication barriers, whether due to language differences, cognitive delays, or cultural factors, can further complicate the diagnosis process. In these situations, healthcare providers may overlook critical symptoms, mistakenly diagnose a condition, or delay treatment. In these situations, wearable technology can collect data and analyze it without bias, which can be critical for helping with a true diagnosis. According to UNICEF data [49], there are approximately 2.415 billion children under the age of 18 globally. In the United States, the population of children stands at 71.5 million, with nearly 33% falling within the 0–5 age range. This demographic necessitates consistent monitoring of developmental milestones, as children in this age group often face challenges in communication, which may impact the accuracy of diagnostic assessments.

There are other developmental disorders that benefit from early detection using wearable devices. Autism spectrum disorder (ASD) is a neurological and developmental disorder affecting the communication, learning ability, and behavioral response of people diagnosed. ASD is a developmental disorder and can be diagnosed at any age, but symptoms appear in the first two years of life. The US Center for Disease Control (CDC) reported that approximately 1 in every 31 children in the U.S. is diagnosed with [50].

In summary, young children often lack the ability to effectively communicate their physical or emotional states, and health monitoring and early intervention are expected to play a critical role in the development of children’s wearable technology. These technologies can enable parents and healthcare professionals to detect potential health issues at an early stage, thereby reducing healthcare costs and improving clinical outcomes. For school-aged children, wearable devices offer not only health-related applications but also potential as educational tools. They can enhance cognitive engagement during learning by providing real-time feedback on attention, activity levels, and emotional states. Additionally, early detection of mental health concerns—particularly among adolescents—may become more feasible through continuous monitoring of physiological and behavioral indicators. Despite the significant benefits that wearable technology may offer for children, robust regulatory frameworks are essential to ensure data privacy and user safety. Clear and enforceable policies can help build trust among parents and guardians, encouraging the responsible adoption of these technologies for the benefit of both individual children and society at large.

## 7. Conclusions: Future Directions and Improvements

Given the need for an effective interface between young children and the healthcare system—particularly due to their limited ability to communicate symptoms—non-invasive wearable devices capable of monitoring and translating physiological data are crucial for enabling early diagnosis and timely intervention. The continuous advancement of artificial intelligence (AI) and machine learning techniques further supports the analysis of complex health data, enhancing the potential for accurate and early detection of medical conditions. However, several limitations must be addressed to optimize the applicability of such devices for pediatric use widely and to facilitate the translation of research innovations into commercially viable and widely adopted technologies. These challenges include the need for technological innovation, improved data collection methods, and robust frameworks to ensure data privacy and security.

### 7.1. Technological Innovations

Technological advancement is crucial for the development of innovative medical devices and diagnostic tools tailored specifically to pediatric populations. Children are not simply smaller versions of adults; they present a distinct set of anatomical and physiological challenges that must be carefully considered during the design and development process. Unlike adults, whose bodies have reached physical maturity, children are in a continuous state of growth and development. This includes significant changes in body size, organ structure, and brain development, which can vary greatly depending on age and developmental stage. As a result, medical devices intended for pediatric use must be designed with a high degree of precision and adaptability to accommodate this variability. Furthermore, the integration of nanotechnology can offer solutions to many of these challenges. By enabling the miniaturization of components—particularly sensors and diagnostic elements—it becomes possible to create devices that are small, lightweight, and non-invasive, which is particularly important for use in infants and young children. These nanoscale technologies can allow for more accurate monitoring of physiological parameters with minimal discomfort, thereby improving both the quality of care and patient compliance.

On the other side of technological advancement, the development of user-friendly applications accessible to both parents and healthcare providers is essential for effective pediatric care. These applications should prioritize intuitive design to ensure ease of use across varying levels of technical proficiency. Moreover, implementing on-device data processing—rather than relying solely on cloud-based solutions (such as Empathica Device)—can significantly enhance data privacy and security, which is particularly important when handling sensitive information related to children. To support advanced functionalities such as real-time analysis and personalized recommendations, the integration of local artificial intelligence (AI) and deep learning models is critical. These locally deployed models not only reduce latency but also minimize the risk of data exposure by keeping sensitive information on the device itself. This approach fosters greater trust among users while maintaining compliance with stringent data protection regulations.

Therefore, a multidisciplinary approach that incorporates technological innovation, developmental biology, and pediatric healthcare expertise is essential to advance the field and ensure that wearable sensors for healthcare applications meet the unique needs of children.

### 7.2. Enhanced Data Collection

Based on the reviewed studies, it is evident that current data collection methods require significant improvement. To address this, future research should focus on developing more comprehensive, accurate, and scalable approaches that can capture real-world, longitudinal data from diverse pediatric populations. Based on the reviewed studies, data collection needs improvement. To enhance the quality and comprehensiveness of data collection in pediatric research, it is essential to consider strategies for involving larger and more diverse cohorts of children. Broadening participation across different age groups, socio-economic backgrounds, and geographic regions can improve the generalizability and relevance of study findings. Moreover, implementing systems for continuous monitoring of daily activities—such as physical movement, sleep patterns, and behavioral indicators—can provide valuable longitudinal data that better reflect children’s real-world experiences. Engaging multiple stakeholders, including parents, guardians, teachers, and healthcare providers, is also critical to ensuring the success of such studies. Their input can inform both the design and implementation of data collection methods, making them more practical, ethical, and child-friendly. Additionally, designing long-term studies requires careful planning around participant retention, ethical considerations, and the sustainability of monitoring technologies. This includes developing flexible protocols that accommodate changes in children’s development over time, and ensuring that data collection remains non-invasive and minimally disruptive to daily life.

### 7.3. Addressing Privacy

Addressing privacy is essential when conducting research or developing technologies that involve children. Due to the sensitive nature of pediatric data and the vulnerability of this population, strict privacy safeguards must be embedded into every stage of data handling. This includes providing a clear and transparent explanation of how data will be collected, processed, stored, and used. Participants and their guardians should have full visibility and control over the data lifecycle, including options for consent, access, and withdrawal. Moreover, systems must be designed to support controlled data recording—ensuring that only relevant, necessary information is captured—and secure data destruction protocols must be established to guarantee that data is permanently deleted when no longer required or upon request. These measures are not only critical for ethical compliance and trust-building with families but also for adhering to legal frameworks such as GDPR, COPPA, or HIPAA, depending on the region. Ensuring privacy and data protection from the outset is fundamental to responsible innovation in child-focused research and technology development.

## Abbreviations

The following abbreviations are used in this manuscript:

ECG	Electrocardiogram
GPS	Global Positioning System
GSM	Global System for Mobile Communications
PPG	Photoplethysmogram
UV	Ultraviolet
OCD	Obsessive–Compulsive Disorder
AI	Artificial Intelligence
EDA	Electrodermal Activity
WES	Wearable Engagement Sensor
IMU	Inertial measurement units
BLE	Bluetooth low-energy
SoC	System on a chip
NIRS	Near-infrared spectroscopy
CCHS	Congenital hypoventilation syndrome
UNICEF	United Nations Children’s Fund
ASD	Autism Spectrum Disorder
CDC	US Center for Disease Control
IRB	Institutional Review Board
FES	Functional Electrical Stimulation
COPPA	Children’s Online Privacy Protection Act
GDPR-K	General Data Protection Regulation for Children
FDA	Food and Drug Administration
MDR	Medical Device Regulation
LENA	Language Environment Analysis
CTC	Conversational Turn Count
VP	Vocal Productivity
AVA	Automated Vocalization Assessment
